# Endoscopic resection of a giant colonic lipoma with endoloop-assisted unroofing technique: A case report

**DOI:** 10.1097/MD.0000000000010995

**Published:** 2018-06-18

**Authors:** Lei Shi, Yuanshun Zhao, Wen Li

**Affiliations:** Department of Endoscopy, Tianjin Union Medical Center, 190, Jieyuan Road, Hongqiao District, Tianjin, China.

**Keywords:** colonic lipoma, endoloop, endoscopic resection, unroofing technique

## Abstract

**Rationale:**

Colonic lipomas are uncommon benign submucosal adipose tumorsthat are usually asymptomatic. Large lipomas can cause symptoms require treatment in principle. We report 1 case of giant colonic lipoma removed with endoloop-assisted unroofing technique instead of conventional surgical bowel resection.

**Patient concerns:**

A 62-year-old female patient presented with intermittent abdominal discomfort for 1 month.

**Diagnosis:**

The patient was diagnosed as having a giant colonic lipoma.

**Intervention:**

Endoscopic resection with endoloop-assisted unroofing technique was performed. On the 22nd day after resection, intestinal obstruction occurred by shedding mass was found; the symptoms of this patient disappeared soon after removal of the mass by endoscopy.

**Outcomes:**

A follow-up colonoscopy 6 months later showed a scarred mucosa at the ligation site and no residual lipoma was observed.

**Lessons:**

Endoscopic resection with endoloop-assisted unroofing technique remains a viable option for giant lipomas; however, postoperative intestinal obstruction caused by shedding mass should be noted.

## 1 Introduction

Colonic lipomas are the most common benign submucosal tumor.^[1]^ They are usually found incidentally during endoscopy, surgery, or autopsy without any symptoms. Some cases may present with symptoms such as abdominal pain, diarrhea, bleeding, obstruction, and intussusception especially when their diameter exceeds 2 cm. The reported size of lipoma varies from 2 mm to 30 cm, with increased likelihood of symptoms as the size is bigger.^[2,3]^ When colonic lipomas become bigger in size or symptomatic they require resection. We report our experience of one case of giant colon lipoma (GCL) that was cleared up after ligation with endoloop and unroofing, together with a literature review.

## 2 Case report

A 62-year-old female patient presented with intermittent abdominal discomfort for 1 month. Her medical history was nonspecific. There was no obvious abnormality in the physical examination. All laboratory examinations including complete peripheral blood cell counts, blood biochemistry, and carcinoembryonic antigen were within normal range. A colonoscopy was performed that showed a giant, sessile, yellowish mass with superficial mucosal necrosis within the transverse colon, which was around 8 cm size with a spindle shape, almost obstructed the colonic lumen. The mass texture is soft and submucosal fat-like tissue was visible when touched by a biopsy forcep. The preoperative diagnosis was colonic lipoma depending on the colonoscopy findings.

**Figure 1:**
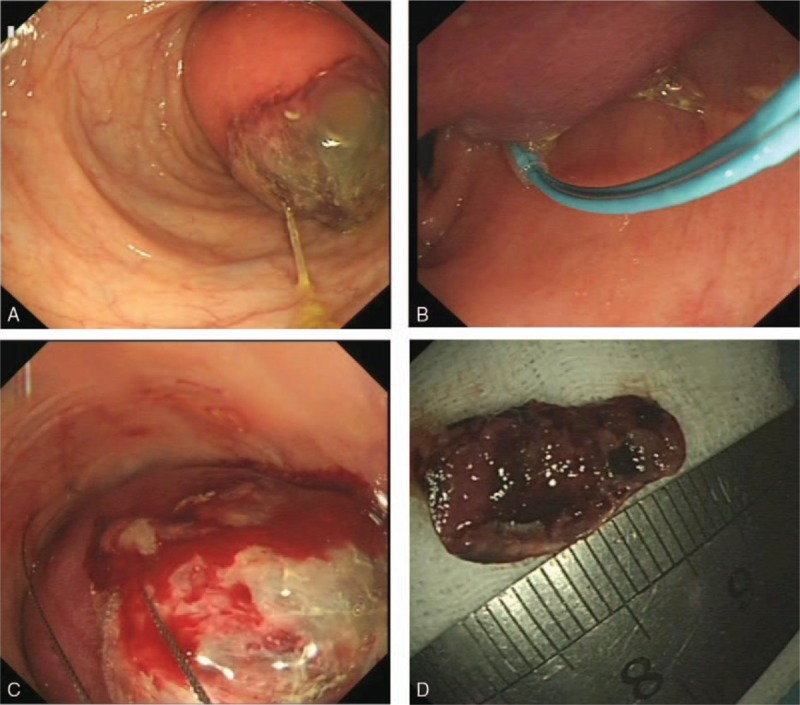
Image of the first treatment. (A) Colonoscopy showed a giant, sessile, yellowish mass with superficial mucosal necrosis within the transverse colon, which was almost obstructing the colonic lumen. (B) The base of the mass was ligatured to block the basal blood supply with endoloop. (C) Unroofing technique was performed with a snare catheter at the top of the lesion. (D) The removed lipoma specimen with partial resection.

Owing to the high risk of complete colonic obstruction and bleeding, we performed endoscopic tumor resection for the patient. We used a ligating device (HX-20U-1; Olympus, Tokyo, Japan) and a detechable endoloop (MAJ-254; Olympus) to ligature the base of the mass to block the basal blood supply; when the endoloop was tightened, the mass color became purple. Then, we performed an unroofing resection with a snare catheter (SD-221U-25; Olympus) at the top of the lesion, so that the remaining lipoma could flow out of the exposed surface (Fig. 1). Pathology of the excised lesion showed the submucosal tumor with characteristic fatty cells, which confirmed the diagnosis of colonic lipoma. Abdominal discomfort of the patient was relieved after resection. Postoperative course was uneventful and was discharged from the hospital on the 3rd postoperative day.

Owing to the giant tumor after resection caused the potential risk of obstruction, intestinal bleeding, and perforation, we informed the patient should pay attention to whether there was blood in the stool and abdominal pain in follow-up.

**Figure 2:**
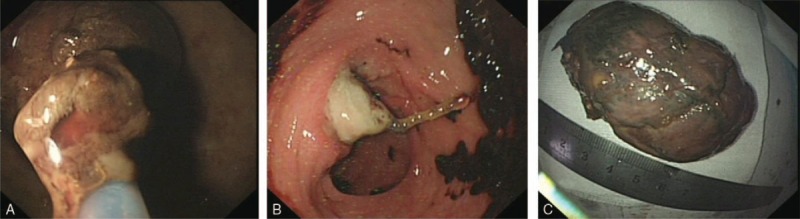
Image of the second treatment on the 22nd day after resection. (A) The shedding mass was stuck in the junction of the descending colon and sigmoid colon. (B) The endoloop in the site of tumor ligation without shedding. (C) The giant shedding mass removed from the intestine lumen.

On the 22nd day after resection, the patient had the abnormal symptoms of abdominal pain, distension, stopping exhaust, and defecation; we considered these discomforts may be caused by intestinal obstruction of the shedding tumor. Therefore, we performed an emergency colonoscopy; a giant mass around 8 cm size was found at the junction of the descending colon and sigmoid colon which blocked the intestine lumen. We removed the mass out of the intestine through the anus with the snare device. The symptoms of this patient disappeared soon after removal of the mass. At the transverse colon, we found the green endoloop in the site of the original tumor ligation without shedding, and we also observed old blood clots within the intestine lumen nearby the wound, but no fresh blood was discharged (Fig. 2). The patient's discomfort was relieved afterwards.

**Figure 3:**
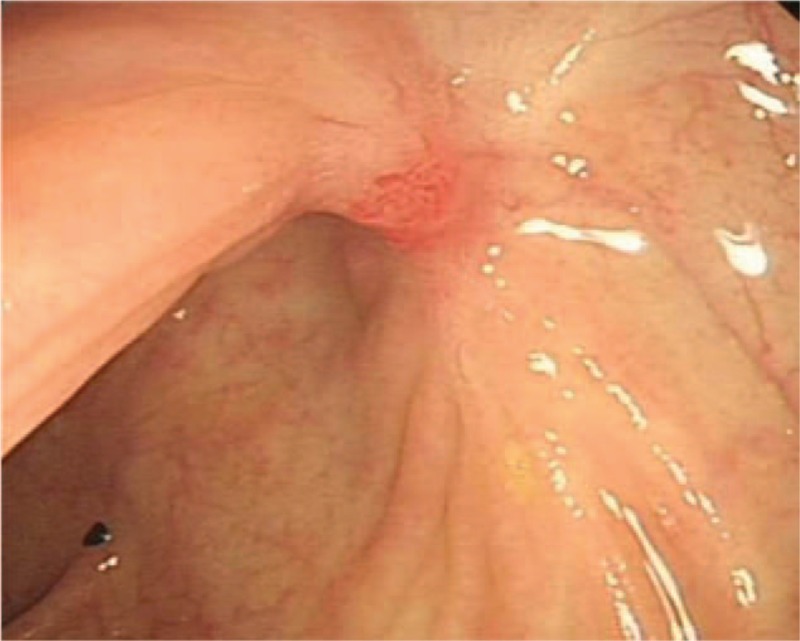
A follow-up colonoscopy 6 months later showed a scarred mucosa without any residual mass.

A follow-up colonoscopy 6 months later showed a scarred mucosa at the ligation site with no lumen stenosis and no residual lipoma (Fig. 3).

The patient signed informed consents. As it is a retrospective case report, ethics approval was not required for this article.

## 3 Discussion

Colonic lipomas are uncommon submucosal nonepithelial tumors; the reported incidence was 0.2% to 4.4%.^[4]^ Colonic lipoma typically presents a smooth, slightly yellow, rounded polyp, with a thick stalk or broad-based attachment, with intact or eroded overlying mucosa. Lipomas can arise in any part of colorectal; however, the most common sites are the cecum, ascending colon, and sigmoid colon, in decreasing frequency. Colonic lipomas originate from the connective tissue of the intestinal walls and most commonly arise from submucosal layer.^[5]^ A colonic lipoma can be diagnosed with barium enema, abdominal computed tomography, endoscopic ultrasound, and colonoscopy. Among these diagnostic tools, colonoscopy can allow fully direct observation of colonic lipomas. Colonoscopy can usually distinguish colonic lipomas from cancer and other neoplasias, especially when the overlying mucosa is intact. Characteristic features include the “tenting sign” (easily elevated mucosa over the lipoma with biopsy forceps), the “pillow sign” (recovery of the indented lipoma with biopsy forceps), and the “naked fat sign” (extrusion of fat tissue after biopsy).^[4,6]^

Cases with symptom or bigger lesion are surgically resected in principle. In general, surgical resection should be performed when the lesion is wide-based or large sessile, cannot distinguish the lipoma from other malignant tumors, causes obvious symptoms, or is involved in the propria muscle or serosal layer.^[3,7–9]^

Endoscopic resection is generally recommended as a safe therapeutic method for lipomas whose diameter is <2 cm, or pedunculated lipomas^[10]^; for these cases, endoscopic resection is considered to be safe with low risk of complications. However, some studies have showed that removal of lipomas >2 cm in diameter is associated with a greater risk of perforation because the adipose tissue is an inefficient conductor for electric current and extend cutting time.^[11]^

With the development of endoscopic techniques, endoscopic resection has become the main treatment of colonic lipomas even if its diameter is >2 cm. Two endoscopic methods to manage lipoma including complete resection and partial resection have been reported.^[12–14]^ The unroofing technique derived from partial resection was first reported by Mimura et al^[12]^ in 1997, which only cuts off the upper half of the submucosal tumor; thus, the risk of complications is reduced. Partial resection is considered to be much safer than complete resection owing to smaller possibility of normal tissue damage and lower chances of complication. For a large size lipoma, remnant lipoma after partialresection could be possible. Therefore, to remove the tumor completely, more resection is more effective.

The endoloop which enables endoscopic ligation of the base of an elevated lesion was first developed by Hachisu^[15]^ in 1991. In recent years, endoloop technique for removal of large colonic lipomas has been described, consisting of looping and ligating the lipoma with the detachable snare.^[16–18]^ By using this technique, the need for electrocautery is avoided, thus eliminating the risk of perforation or bleeding.

In this report, we performed endoloop-assited unroofing technique for a giant sessile lipoma to avoid possible thermal injury. As the base of the mass was too broad to avoid severe snare cautery on the adjacent tissues, while the endoloop was successful in blocking the basal blood supply to promote tumor shedding, the actual resected part was relatively thinner and kept away from the wall of intestinal wall, therefore electric current was well-applied without thermal injury on the adjacent tissues. After the tumor was shedding, the intestinal mucosa could heal itself. At follow-up colonoscopy 6 months later showed scarred mucosa without any remaining lipoma.

Lipomas >2 cm can cause obstruction. In this report, the giant shedding mass was stuck in the junction of the descending colon and sigmoid colon, which caused acute intestinal obstruction. To avoid intestinal perforation caused by distention, emergency colonoscopy should be performed as the patient has symptoms of obstruction after endoloop ligation for giant lipomas.

## 4 Conclusion

This report describes one case presenting with a GCL. Endoscopic resection with endoloop-assisted unroofing technique is safe and effective for giant lipomas, and the lesions could be cleared up spontaneously after the therapy. However, postoperative intestinal obstruction should be noted.

## Author contributions

**Writing – original draft:** Lei Shi, Yuanshun Zhao.

**Writing – review & editing:** Wen Li.
